# Incarcerated femoral hernia containing ovary and fallopian tube in a postmenopausal woman: a rare case report

**DOI:** 10.1093/jscr/rjag149

**Published:** 2026-03-17

**Authors:** Samson Y Murra, Bethelhem B Boba, Eden H Hagos

**Affiliations:** Department of Surgery, College of Health Science, Addis Ababa University, Zambia Street, Addis Ababa, Ethiopia, 5657, Ethiopia; Department of Surgery, College of Health Science, Addis Ababa University, Zambia Street, Addis Ababa, Ethiopia, 5657, Ethiopia; College of Health Science, Addis Ababa University, Zambia Street, Addis Ababa, Ethiopia, 5657, Ethiopia

**Keywords:** femoral hernia, incarcerated hernia, ovary, fallopian tube, postmenopausal woman

## Abstract

Femoral hernias account for a small proportion of groin hernias but occur more frequently in women and elderly individuals. Herniation of adnexal structures such as the ovary and fallopian tube is extremely rare, particularly in postmenopausal women. A 70-year-old postmenopausal woman presented with a 2-year history of a left groin swelling that became painful and irreducible 12 h prior to admission. Preoperative ultrasonography suggested an incarcerated inguinal hernia containing bowel loops. Adnexal involvement was not suspected preoperatively, reflecting the diagnostic challenge. Emergency surgical exploration revealed a left femoral hernia containing a viable ovary and fallopian tube, representing an unexpected intraoperative finding. The adnexal structures were reduced, and the femoral defect was repaired using a tissue-based repair. The postoperative course was uneventful. Femoral hernias containing adnexal structures in postmenopausal women are exceptionally rare and often diagnosed intraoperatively. Awareness of this possibility, prompt surgical intervention, and careful intraoperative assessment are essential.

## Introduction

Hernias represent a major global health burden, with an estimated 32.5 million cases worldwide as of 2019 [1]. Groin hernias in women are considerably less common than in men [2]. However, femoral hernias constitute a disproportionately large share of female groin hernias and occur 4–5 times more frequently in women than in men [2, 3]. Age is a major determinant of risk, making older women particularly vulnerable [2, 4].

Typical femoral hernia contents include omentum or small bowel, but adnexal structures such as the ovary may rarely be involved [5–7]. Ovarian herniation varies markedly by age: among reproductive-aged and adolescent females, ovaries are involved in roughly 30% of groin hernias, whereas in infants and children, the incidence rises to ~71%, largely due to a persistent canal of Nuck [7, 8]. In contrast, ovarian or tubal herniation in postmenopausal women is exceptionally uncommon and typically reported as isolated case reports.

Preoperative recognition of adnexal herniation is difficult due to nonspecific clinical features [9]. Ultrasonography, with attention to follicular morphology and color Doppler, is the preferred initial modality to identify ovarian tissue and assess viability [9, 10]. Computed tomography may be used if ultrasound is inconclusive [6, 11]. However, pain, patient habitus, and operator dependency may limit ultrasonographic sensitivity, particularly in emergency settings, and the diagnosis is frequently made only at surgery.

Definitive management of incarcerated femoral hernia involves surgical exploration, reduction of viable contents, and repair of the femoral defect [5, 12]. Mesh repair is standard for elective cases, but tissue-based repairs are often selected in the emergent setting when contamination risk or diagnostic uncertainty exists. When adnexal structures are involved, careful assessment of viability guides the decision between preservation and resection.

We present a rare case of an elderly woman with an incarcerated femoral hernia containing a viable ovary and fallopian tube.

## Case report

A 70-year-old postmenopausal woman presented with a 2-year history of a left groin swelling that had been reducible until 12 h prior to her current presentation, when it became irreducible and was associated with groin pain and non-bilious vomiting. She denied fever, abdominal distension, constipation, or obstipation. There was no history of chronic cough, prior abdominal surgery, or known chronic medical illness. The patient appeared acutely ill, but her vital signs were within normal limits. A firm, tender, irreducible mass measuring ~3 × 4 cm was noted in the left groin region without overlying skin discoloration. The remainder of the abdominal and systemic examinations was unremarkable. Laboratory investigations were within normal ranges. Abdominopelvic ultrasonography demonstrated a 1.6-cm defect in the left inguinal region containing bowel loops with preserved peristalsis and normal Doppler flow. The hernia was non-reducible with gentle probe pressure, consistent with an incarcerated inguinal hernia.

**Figure 1:**
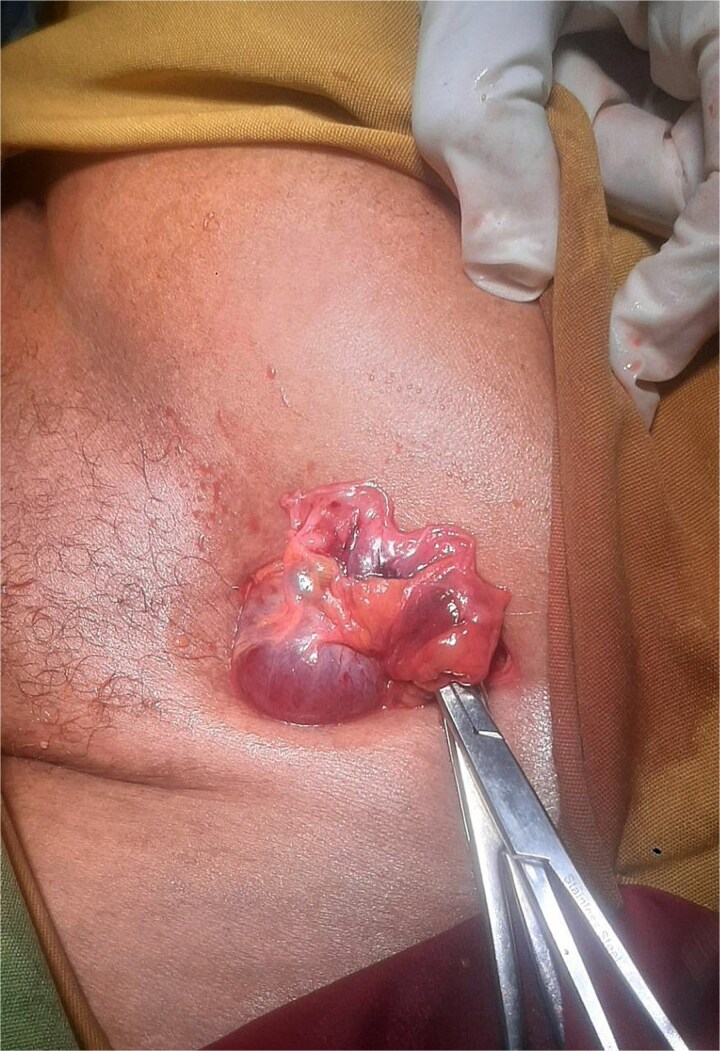
Intraoperative photograph demonstrating a left femoral hernia sac opened through a left inguinal incision, revealing a viable ovary and fallopian tube without evidence of ischemia or torsion, prior to reduction.

With a preoperative diagnosis of incarcerated left inguinal hernia, the patient underwent emergency surgery under spinal anesthesia. Surgical exploration was performed through a left inguinal oblique incision. Intraoperatively, a left femoral hernia containing a viable ovary and fallopian tube was identified (Fig. 1). The adnexal structures were carefully assessed and found to be viable, with no evidence of ischemia or torsion. The adnexal structures were then reduced into the peritoneal cavity, and the femoral defect was repaired using a tissue-based McVay repair, selected due to the emergency setting. Postoperatively, the patient received multimodal analgesia and was initiated on oral feeding six hours after surgery. Her postoperative course was uneventful, and she was discharged on the fifth postoperative day. Follow-up at one week and one month revealed a smooth recovery. Postoperative gynecologic consultation confirmed no need for further intervention.

## Discussion

Femoral hernias account for only 2%–4% of all groin hernia repairs, yet they have a strong female predominance [2]. Ovarian herniation in adults, whether inguinal or femoral, is exceedingly uncommon and primarily described in isolated case reports; only a small number of adult cases have been published in recent years [10, 12]. This contrasts sharply with pediatric patients, in whom ovarian involvement is relatively common [7, 8]. The occurrence in postmenopausal women is especially unusual, likely reflecting ovarian atrophy and reduced ligamentous laxity with age.

Diagnosis is challenging. Clinically, incarcerated femoral hernias with ovarian content resemble other incarcerated groin hernia presentations, including groin pain, a firm irreducible mass, and occasional vomiting without specific gynecologic features [9]. Ultrasonography with color Doppler is the first-line imaging modality in women, as it can demonstrate follicular architecture and vascular flow suggestive of an ovary [9, 10]. However, adnexal structures may not be recognized preoperatively due to technical limitations, acute pain, or operator dependency. Computed tomography can provide clearer anatomic detail when ultrasound is inconclusive; however, many patients proceed directly to emergency surgery without cross-sectional imaging, particularly in resource-limited settings like ours.

The surgical approach depends on the clinical presentation and viability of the hernia contents. Incarcerated hernias are typically explored through an inguinal approach, whereas strangulated hernias may require laparotomy. In this case, surgery was performed via an inguinal incision based on the preoperative diagnosis. Careful intraoperative dissection is essential to avoid injury to the ovarian vascular pedicle. When adnexal structures are viable, reduction and preservation are recommended, as delayed intervention may result in torsion, infarction, and loss of the organ [5, 10]. In our case, the left ovary and fallopian tube were viable and were successfully preserved. Similar reports describe preserved ovarian tissue after repair of femoral or inguinal hernias [9].

Repair of the femoral defect may be performed using mesh-based or tissue-based techniques. Although mesh repair offers the lowest recurrence rates and is generally recommended for most elective procedures, tissue-based repair was chosen in this emergency setting due to the acute presentation and concern for potential contamination, in accordance with the surgeon's judgment, thereby avoiding potential mesh-related complications. Femoral hernias have a significantly higher risk of incarceration and strangulation compared with inguinal hernias, making timely diagnosis and repair essential [13]. Our patient’s uneventful recovery reflects early intervention.

This case underscores three key points: adnexal herniation should remain in the differential diagnosis of a groin mass even in elderly patients; ultrasonography with Doppler can assist in preoperative identification, but it may be inconclusive; and timely surgical exploration with preservation of viable adnexal structures and definitive hernia repair results in an excellent outcome.
